# Depression, Anxiety, and Health-Related Quality of Life in Adults with Type 2 Diabetes

**DOI:** 10.3390/jcm13206028

**Published:** 2024-10-10

**Authors:** Monira Alwhaibi

**Affiliations:** 1 Department of Clinical Pharmacy, College of Pharmacy, King Saud University, Riyadh 11149, Saudi Arabia; malwhaibi@ksu.edu.sa; Tel.: +966-535-384-152; 2 Medication Safety Research Chair, College of Pharmacy, King Saud University, Riyadh 11437, Saudi Arabia

**Keywords:** anxiety, depression, mental illnesses, diabetes, quality of life, SF-12

## Abstract

**Background:** Adults with type 2 diabetes are at a greater probability of suffering from mental health issues, which could result in a substantial effect on their HRQoL (health-related quality of life). Thus, the focus of this research was to investigate the relationship between comorbid anxiety, depression, and HRQoL among individuals with type 2 diabetes. **Methods:** Data from the Medical Expenditure Panel Survey from 2016 to 2021 were used to identify adult patients with type 2 diabetes diagnoses for this research. The MEPS used the SF-12 to measure HRQoL. The study employed multivariable linear regression to analyze the association between anxiety, depression, and HRQoL in individuals with type 2 diabetes, taking into consideration several confounding variables such as age, gender, and comorbidities. **Results:** This study included 5259 individuals with type 2 diabetes, of which the mean age was 52.9 years, 50.7% were men, and 49.3% were women. The findings from this sample show a negative relationship between anxiety, depression, and type 2 diabetic patients’ HRQoL. It shows that after adjusting for other factors, diabetes patients with depression (MCS: = −6.817), anxiety (MCS: = −4.957), and both (MCS: = −0.344) have a significantly poorer HRQoL than those without these mental health comorbidities (*p*-value < 0.001). It also demonstrated the adverse effects on HRQoL of having coexisting chronic illnesses like heart disease, hypertension, and other chronic illnesses, along with a low socioeconomic status. Additionally, it revealed the benefits of employment, education, and regular exercise for HRQoL. **Conclusions:** The study’s findings highlight the links between anxiety, depression, and type 2 diabetes patients’ poor HRQoL. It also showed the adverse effects of coexisting chronic diseases and low socioeconomic status on HRQoL and the benefit of work and exercise on HRQoL. These findings can help policymakers to reform healthcare and enforce the early detection and treatment of anxiety and depression to enhance the HRQoL of type 2 diabetic patients.

## 1. Introduction

Diabetes is a chronic and highly prevalent health condition. The global diabetes prevalence is projected to increase to 439 million adults (7.7% prevalence) by 2030 [1]. In the United States (US), the number of adults with diabetes is projected to increase from 26.8 million adults in 2010 to 36.0 million in 2030 [1]. Diabetes is one of the leading causes of premature death globally [2]. Nearly 50% of those with diabetes die from cardiovascular disease (CVD), and 10% die from kidney failure. It is a costly chronic illness; the estimated cost of diabetes worldwide was USD 966 billion in 2021 and is predicted to increase to USD 1054 billion by 2045 [3].

Type 2 diabetes has been associated with several illnesses [4] and has a substantial negative impact on a person’s cognitive and functional abilities and their health-related quality of life (HRQoL) [2,5,6]. Individuals with type 2 diabetes suffer more from anxiety and depression than those without diabetes [7,8,9,10,11]. Among diabetic patients, 12–19% experienced depression [8,9,11] and 14–28% had generalized anxiety disorder (GAD) [7,10]. According to Cannon et al. (2018), 41% of individuals with poor glycemic control had low psychological well-being, such as depression and anxiety, which has a detrimental impact on HRQoL [5]. There is a bidirectional relationship between depression and anxiety disorders and type 2 diabetes [7,12,13,14]. There is a higher risk of developing depression and anxiety in patients with type 2 diabetes and vice versa, which has been attributed to the influence of depressive symptoms on adult behaviors, such as inactive lifestyles, smoking, and metabolic disturbances [12].

Patients with type 2 diabetes who also have coexisting anxiety or depression are more likely to have a higher risk of diabetes-related complications, healthcare cost and utilization, chance of disability and lost productivity, risk of death, and worse glycemic control [8,15,16,17,18]. A retrospective analysis data conducted on 16,159 patients using national data found that those with diabetes and depression had higher rates of medical visits and a worse physical function compared to those without these illnesses [19]. In addition, depression or anxiety comorbidity impaired type 2 diabetes sufferers’ health-related quality of life (HRQOL) [20,21,22,23,24,25,26]. In an observational study of 537 adult outpatients with diabetes, depression symptoms were negatively associated with all eight dimensions of the 36-item Short Form Survey (SF-36), even after controlling for other covariates [20].

Health-related quality of life, which pertains to an individual’s functioning and perceived physical, mental, and social well-being, is a crucial outcome variable for diabetes patients [27]. Maintaining a positive HRQoL in the face of the challenges posed by diabetes is one of the main goals for those with it. Therefore, finding the root causes of a lowered HRQoL is fundamental, especially in light of mental health comorbidities. There are no published population-based data in the US about the psychological comorbidities related to HRQoL in adults with diabetes. Furthermore, there is a shortage of global studies investigating the effects of psychological comorbidities on the HRQoL associated with diabetes. Also, most of the published studies have used self-reported measures of depression and anxiety among a small sample size [26]. Therefore, the aim of this study is to determine the relationship between anxiety and depression and HRQoL in a representative sample of type 2 diabetic adults in the US population.

## 2. Methods

### 2.1. Study Design and Data

The study was retrospective and cross-sectional. This study used national US data from the Medical Expenditure Panel Survey (MEPS) from 2016 to 2021. The MEPS gathers health-related information and other data from the non-institutionalized US civilian population.

### 2.2. Study Population

The inclusion criteria in this study were as follows: adults aged 18 to 64 diagnosed with type 2 diabetes that were alive, and adults with no missing information in relation to HRQoL data. Individuals with type 2 diabetes were identified using the clinical diagnostic codes from the International Classification of Diseases, tenth Revision, Clinical Modification (ICD-10-CM), which are included in the MEPS medical conditions (icd10cdx for Type 2 Diabetes Mellitus is ‘E11’).

### 2.3. Measures

#### 2.3.1. Study Outcome: Health-Related Quality of Life

This survey is a generic evaluation of an individual or patient’s health-related quality of life. Adults who participated in the survey and were at least 18 years old had to complete the self-administered survey to determine their HRQoL. To determine patients’ HRQoL, the MEPS collects this information using the Short-Form 12 Version 2 (SF-12V2) questionnaire. The SF−12v2 is a valid and reliable instrument in diabetic patients of the US population [28,29]. The SF-12v2 Health Survey (12 items) is an abbreviated version of the SF-36v2 (36 items). The SF-36v2 measures eight domains, which are also addressed in the SF-12v2; however, because of the assessment’s conciseness, the SF-12v2 has only two domains—(1) Physical Component Summary (PCS) and (2) Mental Component Summary (MCS) of [28]—have a range of 0 to 100, where higher scores in these domains indicate a better physical and mental HRQoL.

#### 2.3.2. Independent Variables

Adults with type 2 diabetes were categorized into four mutually exclusive categories (diabetes only, diabetes and depression, diabetes and anxiety, and diabetes and both mental illnesses). Depression and anxiety were identified from the MEPS using the ICD-10-CM codes for depression (icd10cdx: ‘F34’,‘F39’,‘F32’) and anxiety (icd10cdx: ‘F40’, ‘F41’).

Other independent variables included sociodemographic characteristics (i.e., gender, age group (18–39, 40–49, and 50–64), race/ethnicity (White, African American, Latino, and others), marital status (married, widowed, separated/divorced, and never married), the region of residence (northeast region, mid-west region, south region, and west region), education level (less than high school, high school, and greater than high school), health insurance (private insurance, public insurance, and uninsured), medication insurance (have medication insurance and no medication insurance), employment status, and poverty status). Poverty status was defined using family income in relation to the federal poverty line (FPL) and was classified into poor (less than 100% FPL), near poor (100% to less than 200% FPL), middle income (200% to less than 400% FPL), and high income (greater or equal to 400% FPL). Other independent variables included physical activity (three times a week or more/less than three times a week), perceived physical health (excellent/very good, good, and fair/poor), and comorbid chronic health conditions identified in the MEPS using the ICD-10-CM codes.

### 2.4. Statistical Analyses

Chi-square tests were used to determine significant differences in baseline characteristics between diabetes groups, in which all are categorical variables. Variations in HRQoL between diabetes groups were identified using analysis of variance (ANOVA), followed by Tukey’s post hoc test for multiple comparisons to find out which group means differ from one another. Statistical significance was defined as a *p*-value of less than 0.05. Multivariable linear regression was used after adjustment for all independent factors to evaluate the relationship between diabetes groups and HRQoL. We adjusted for variance weights (strata and primary sample unit) from the MEPS, which was reflected in the statistical analyses to account for all estimations. The data were analyzed using SAS 9.4 (SAS Institute Inc., Cary, NC, USA).

## 3. Results

### 3.1. Sample Description

Table 1 presents the characteristics of the study sample; a total of 5259 individuals with type 2 diabetes were included in this study. Adults aged between 50 and 64 years old (68.7%), and those who had a “greater than high school” education level (83.3%) made up the majority of the study sample. Around 10% of adults with type 2 diabetes had depression, 8% had anxiety, and 7% had both conditions.

Women with type 2 diabetes experienced a higher percentage of depression (13.0% vs. 6.5%) and anxiety (10.4% vs. 6.5%; *p*-value < 0.0001) than men did. Additionally, diabetic patients who were unemployed had a higher percentage of depression (14.0% vs. 7.2%) and comorbid anxiety and depression (12.0% vs. 4.2%; *p*-value < 0.0001) than employed adults with diabetes. Additionally, diabetic adults with chronic illnesses (e.g., hypertension, hyperlipidemia, asthma, and arthritis) are considerably more likely to suffer from depression and anxiety than diabetic adults without these illnesses (*p*-value < 0.0001).

### 3.2. Health-Related Quality of Life Scores by Diabetes Categories

The differences between the type 2 diabetes categories in the PCS and MCS scores for HRQoL are displayed in Table 2. A comparison between different diabetes groups was performed using the ANOVA test, which revealed a significant difference in all groups (*p*  <  0.05), followed by Tukey’s post hoc test for multiple comparisons, which found the following.

Regarding PCS score, patients with diabetes only have a significantly higher mean PCS score as compared to the other groups (diabetes only, diabetes and depression, and diabetes and anxiety). Patients with diabetes and depression have a significantly lower mean PCS score as compared to the diabetes only group but not the other groups. Patients with diabetes and anxiety have a considerably higher mean PCS score as compared to those with diabetes and depression. Patients with diabetes and anxiety/depression have a significantly lower mean PCS score as compared to those with diabetes only or those with diabetes and depression.

Regarding MCS score, patients with diabetes only have a significantly higher mean MCS score as compared to the other groups (diabetes only, diabetes and depression, and diabetes and anxiety). Patients with diabetes and depression have a significantly lower mean MCS score as compared to the diabetes only group and the diabetes and anxiety group, but have a higher mean MCS score as compared to the diabetes and anxiety/depression group. Patients with diabetes and anxiety have a significantly lower mean MCS score as compared to those with diabetes only, but a higher mean MCS score as compared to the diabetes and depression group and the diabetes and anxiety/depression group. Patients with diabetes and anxiety/depression have a significantly lower mean MCS score as compared to all other groups.

### 3.3. Factors Associated with HRQoL in Patients with Type 2 Diabetes from Multivariable Adjusted Linear Regression Analysis

Table 3 demonstrates a key finding from our study. It shows that after adjusting for other factors, type 2 diabetes patients with depression (MCS: = −6.817), anxiety (MCS: = −4.957), and both (MCS: = −0.344) have a significantly poorer HRQoL than those without these mental comorbidities (*p*-value < 0.001).

Factors negatively related to HRQoL include poverty status, region of residence, and concurrent comorbidities, including heart disease, hypertension, hyperlipidemia, asthma, COPD, arthritis, and GERD. For example, those with a poor income had a worse HRQoL for both their physical health summary score (PCS: = −1.902; *p*-value < 0.001) and their mental health summary score (MCS: = −1.294; *p*-value < 0.001) compared to those with a high income.

Factors positively related to HRQoL included gender, education level, being employed, general health, and medication insurance. For instance, diabetic patients who were employed had a higher HRQoL than those who were unemployed in both physical health (PCS: = 5.036; *p*-value < 0.001) and mental health (MCS: = 2.534; *p*-value < 0.001).

## 4. Discussion

This study evaluated the relationship between type 2 diabetes patients’ mental comorbidities and their HRQoL. According to our study findings, comorbidities of anxiety and depression among adults with type 2 diabetes are associated with a poor HRQoL compared to those without these comorbidities. This result lines up with other previous research showing a reciprocal relationship between anxiety and HRQoL as well as depression and HRQoL in adults with type 2 diabetes [20,21,22,23,24,25].

Our study also highlights the profound influence of socioeconomic factors on HRQoL. For example, in relation to poverty status, those with a low income have a lower HRQol. This finding is consistent with prior studies that reported that poor economic status was significantly associated with a poor HRQOL [30,31]. Income is a key indicator of social class; a higher income has been linked to better health and fewer health risks, whereas a lower income is linked to increased exposure to risk factors for disease development [32]. Our research reveals a negative link between being medically insured and HRQoL, which is in contrast to the findings of of Bharmal et al., who have demonstrated that adults lacking health insurance had a significantly lower HRQoL (i.e., lower Physical and Mental Component Scores) than the insured [33]. Additionally, diabetic patients’ HRQoL was shown to be adversely associated with hypertension, heart disease, hyperlipidemia, COPD, asthma, arthritis, and GERD. According to earlier research, chronic disorders are linked to a poor HRQoL because of their chronic nature and disease management [31,34,35,36]. Therefore, improving HRQoL has been recognized as a noteworthy outcome and an indicator of the beneficial effects of disease management.

The present investigation has also revealed that several characteristics, such as marital status, employment, perceived general health, and physical activity, positively relate to HRQoL; these findings align with prior research among individuals with other illnesses [24,37,38,39]. Physical activity has been reported to be positively associated with HRQoL by some published studies in the diabetic adult population [20,40]. Lifestyle interventions positively impact depression symptoms in adults with diabetes. For example, a multi-site randomized controlled trial (Look AHEAD trial) that involved 5145 overweight or obese people with type 2 diabetes found that intensive lifestyle interventions can reduce depressive symptoms compared to control interventions [41].

### 4.1. Strengths and Limitations

This study used a nationwide representative sample of US adults with type 2 diabetes to examine how depression and anxiety are related to diabetic adults’ HRQoL, whereas previously published studies [20,21,22,23] were conducted in a single center with a small sample size. Furthermore, wide ranges of confounding variables that may affect the association between depression, anxiety, and HRQoL have been taken into account. These variables include socioeconomic characteristics, treatment accessibility, physical activity, and medical comorbidities. In addition, we measured the impact of several common comorbidities among diabetic patients; therefore, we have overcome previous studies that have combined all comorbidities rather than measuring their effect separately on HRQoL; however, it is essential to consider these limitations when appraising the study’s results. For example, the MEPS does not provide data on the diabetic complications, severity, glycemic control, or duration of diabetes, which have been shown to be related to a poor HRQoL in individuals with diabetes [30,40,42]; therefore, they were not adjusted for in the regression analysis. Additionally, the study used a cross-sectional design; thus, establishing a causal relationship is impossible.

### 4.2. Policy, Practice, and Public Implications

The evidence conveyed in this study can be used by policymakers to advance healthcare planning and resource provision to improve mental health issues and quality of life for individuals with type 2 diabetes. Additionally, this study highlights that healthcare providers should view type 2 diabetes management holistically, and should provide screening and early treatment for mental health conditions to enhance diabetic adults’ HRQoL [6]. This study implies that public health, by endorsing physical exercise, can importantly lessen the mental health issues’ severity and improve the HRQoL of adults with diabetes.

## 5. Conclusions

The study’s findings provide insight into the links between type 2 diabetes patients’ impaired HRQoL and their anxiety and depression. It also illustrated the detrimental effects of chronic diseases and low socioeconomic status on HRQoL and the beneficial effects of work and exercise on HRQoL. Numerous health implications of this study include improved healthcare, resource allocation, and the promotion of lifestyle changes to enhance the HRQoL of type 2 diabetic patients.

## Figures and Tables

**Table 1:** Study sample characteristics (*n* = 5259). Characteristics by diabetes groups among adults with type 2 diabetes. Medical Expenditure Panel Survey 2016–2021.

		Total Sample		Diabetes Only		Diabetes and Depression		Diabetes and Anxiety		Diabetes and Depression and Anxiety		
		*n*	wt.%	*n*	wt.%	*n*	wt.%	*n*	wt.%	*n*	wt.%	*p*-Value
All		5259	100.0	3921	74.9	528	9.7	419	8.4	391	7.0	
Age in years												
	18–39	585	12.5	432	75.9	54	7.8	49	8.1	50	8.2	0.580
	40–49	972	18.8	729	74.5	95	9.7	73	7.4	75	8.3	
	50–64	3702	68.7	2760	74.8	379	10.0	297	8.7	266	6.5	
Gender												
	Women	2846	49.3	1945	67.2	360	13.0	266	10.4	275	9.5	<0.0001
	Men	2413	50.7	1976	82.4	168	6.5	153	6.5	116	4.7	
Race/ethnicity												
	White	2383	55.2	1561	67.4	310	12.0	257	10.9	255	9.7	<0.0001
	African American	1081	16.2	889	83.8	82	6.9	63	5.7	47	3.5	
	Latino	1300	17.9	1064	84.4	89	5.9	73	5.4	74	4.4	
	Others	495	10.6	407	84.0	47	8.5	26	4.6	15	2.9	
Marital status												
	Married	2638	54.7	2101	79.6	222	8.5	192	7.2	123	4.7	<0.0001
	Wid./Sep./Div.	1559	26.5	1051	66.8	193	11.9	146	10.5	169	10.7	
	Never married	1061	18.8	768	72.5	113	10.1	81	9.0	99	8.5	
Education level												
	<HS	431	5.3	341	80.6	36	8.0	29	7.8	25	3.6	0.378
	HS	709	10.3	543	75.3	62	8.4	50	7.7	54	8.6	
	>HS	4047	83.3	2977	74.3	425	10.0	336	8.6	309	7.1	
Region												
	Northeast	703	14.0	522	76.2	70	9.0	46	7.3	65	7.4	0.015
	Mid-west	1163	23.5	796	69.9	160	12.6	91	7.8	116	9.7	
	South	2268	42.4	1728	75.5	194	8.8	209	10.0	137	5.7	
	West	1125	20.1	875	78.5	104	8.7	73	6.3	73	6.4	
Employment												
	Employed	2967	63.6	2437	81.8	219	7.2	188	6.7	123	4.2	<0.0001
	Not employed	2289	36.4	1481	62.7	309	14.0	231	11.3	268	12.0	
Poverty status												
	Poor	1208	16.4	778	61.1	163	13.7	126	12.2	141	13.0	<0.0001
	Near Poor	1129	17.8	825	72.7	113	10.8	73	6.8	118	9.7	
	Middle Income	1438	28.1	1127	76.8	122	8.3	119	9.6	70	5.3	
	High Income	1484	37.7	1191	80.5	130	8.5	101	6.6	62	4.5	
Health Insurance												
	Private	2913	64.6	2329	79.3	250	8.5	199	7.3	135	4.9	<0.0001
	Public	2002	30.2	1288	63.4	261	13.2	208	11.0	245	12.4	
	Uninsured	344	5.2	304	86.3	17	4.1	12	6.8	11	2.8	
Rx Insurance												
	Rx insurance	2543	57.2	2033	79.3	218	8.3	180	7.7	112	4.8	<0.0001
	No Rx insurance	2716	42.8	1888	69.0	310	11.6	239	9.3	279	10.1	
General health												
	Excellent/very good	1275	26.7	1078	84.3	96	7.2	63	5.8	38	2.7	<0.0001
	Good	2059	40.2	1625	78.4	160	8.0	158	7.6	116	6.0	
	Fair/poor	1925	33.2	1218	63.0	272	13.8	198	11.5	237	11.8	
Physical activity												
	3/week	2102	40.8	1677	80.1	178	7.7	131	6.5	116	5.7	<0.0001
	No exercise	3141	59.0	2230	71.2	349	11.1	287	9.7	275	8.0	
Heart												
	Yes	733	13.5	476	69.0	104	13.2	79	9.3	74	8.4	<0.0001
	No	4526	86.5	3445	75.8	424	9.1	340	8.2	317	6.8	
Hypertension												
	Yes	3200	58.3	2279	71.3	374	11.8	283	9.3	264	7.6	<0.0001
	No	2059	41.7	1642	79.9	154	6.8	136	7.1	127	6.2	
Hyperlipidemia												
	Yes	2759	51.5	1925	70.7	322	11.5	245	9.2	267	8.6	<0.0001
	No	2500	48.5	1996	79.3	206	7.7	174	7.6	124	5.4	
Asthma												
	Yes	715	12.1	404	56.1	104	14.2	105	16.5	102	13.3	<0.0001
	No	4544	87.9	3517	77.5	424	9.1	314	7.3	289	6.2	
COPD												
	Yes	398	7.4	207	50.1	59	16.6	58	16.0	74	17.3	<0.0001
	No	4861	92.6	3714	76.8	469	9.1	361	7.8	317	6.2	
Arthritis												
	Yes	848	15.5	471	56.0	149	17.5	99	12.7	129	13.7	<0.0001
	No	4411	84.5	3450	78.3	379	8.3	320	7.6	262	5.8	
GERD												
	Yes	730	13.1	393	55.1	129	16.8	93	14.2	115	14.0	<0.0001
	No	4529	86.9	3528	77.9	399	8.6	326	7.5	276	6.0	

COPD: chronic obstructive pulmonary disease; GERD: gastroesophageal reflux disease; HS: high school; Rx: medication insurance; Wid./Sep./Div.

**Table 2:** Means and standard errors for HRQoL scores by type 2 diabetes group.

		Total Sample		Diabetes Only		Diabetes and Depression		Diabetes and Anxiety		Diabetes and Depression and Anxiety		
		Mean	SD	Mean	SE	Mean	SE	Mean	SE	Mean	SE	*p*-Value
HRQoL												
	PCS	42.27	12.00	44.92	0.28	38.26	0.79	40.26	0.79	36.78	0.95	<0.0001
	MCS	48.65	10.70	51.73	0.20	42.69	0.61	44.63	0.78	37.81	0.77	<0.0001

Results represent significant weighted mean differences by diabetes groups using ANOVA tests followed by Tukey’s post hoc test. MCS stands for Mental Component Summary; PCS stands for Physical Component Summary; SE stands for standard error; and SD stands for standard deviation.

**Table 3:** Multivariable adjusted linear regressions on health-related quality of life among adults with type 2 diabetes; MEPS 2016–2021.

		Health-Related Quality of Life					
		Physical Component Summary			Mental Component Summary		
		Regression Coefficients	SE	*p*-Value	Regression Coefficients	SE	*p*-Value
Diabetes Group							
	DM and depression	−1.486	0.078	<0.0001	−6.817	0.012	<0.0001
	DM and anxiety	0.180	0.074	0.0160	−4.957	0.064	<0.0001
	DM and depression and anxiety	−1.067	0.066	<0.0001	−10.344	0.116	<0.0001
	DM only (Ref.)						
Age in years							
	18–39	3.904	0.022	<0.0001	−2.173	0.028	<0.0001
	40–49	1.096	0.039	<0.0001	−0.619	0.019	<0.0001
	50–64 (Ref.)						
Gender							
	Women	0.404	0.057	<0.0001	0.173	0.012	<0.0001
	Men (Ref.)						
Race/ethnicity							
	White	0.759	0.104	<0.0001	0.864	0.027	<0.0001
	African American	1.078	0.027	<0.0001	0.227	0.033	<0.0001
	Latino	0.183	0.032	<0.0001	−0.763	0.024	<0.0001
	Others (Ref.)						
Marital status							
	Married	−0.136	0.022	<0.0001	1.167	0.052	<0.0001
	Widow/Separated/Divorce	0.468	0.024	<0.0001	1.626	0.050	<0.0001
	Never married (Ref.)						
Education level							
	>High school	−2.015	0.055	<0.0001	1.035	0.029	<0.0001
	High school	−1.843	0.070	<0.0001	0.681	0.048	<0.0001
	<High school (Ref.)						
Region							
	Northeast	0.816	0.015	<0.0001	−0.164	0.018	<0.0001
	Mid-west	0.008	0.016	0.6100	−0.483	0.008	<0.0001
	South	−0.730	0.040	<0.0001	−0.438	0.021	<0.0001
	West (Ref.)						
Employment							
	Employed	4.976	0.074	<0.0001	2.319	0.022	<0.0001
	Not employed (Ref.)						
Poverty status							
	Poor	−1.902	0.080	<0.0001	−1.294	0.038	<0.0001
	Near Poor	−1.036	0.057	<0.0001	−0.844	0.048	<0.0001
	Middle Income	−0.699	0.031	<0.0001	0.563	0.021	<0.0001
	High Income (Ref.)						
Health Insurance							
	Private	0.414	0.064	<0.0001	0.361	0.088	<0.0001
	Public	−2.608	0.017	<0.0001	−1.317	0.122	<0.0001
	Uninsured (Ref.)						
Medication Insurance							
	Medication insurance	0.165	0.065	0.0113	0.050	0.111	<0.0001
	No insurance (Ref.)						
General health							
	Excellent/very good	10.780	0.076	<0.0001	6.365	0.041	<0.0001
	Good	6.830	0.019	<0.0001	4.134	0.047	<0.0001
	Fair/poor (Ref.)						
Physical activity							
	3 times per week	1.649	0.060	<0.0001	0.668	0.015	<0.0001
	No exercise (Ref.)						
Heart							
	Yes	−3.317	0.036	<0.0001	−0.391	0.075	<0.0001
Hypertension							
	Yes	−0.712	0.065	<0.0001	0.364	0.012	<0.0001
Hyperlipidemia							
	Yes	−0.336	0.004	<0.0001	−0.478	0.031	<0.0001
Asthma							
	Yes	−2.086	0.045	<0.0001	−1.524	0.049	<0.0001
COPD							
	Yes	−1.867	0.066	<0.0001	1.008	0.038	<0.0001
Arthritis							
	Yes	−3.250	0.049	<0.0001	−0.877	0.048	<0.0001
GERD							
	Yes	−0.916	0.087	<0.0001	0.520	0.069	<0.0001

COPD: chronic obstructive pulmonary disease; GERD: gastroesophageal reflux disease; Ref: reference group; SE: standard error.

## Data Availability

Researchers can access the dataset used in this study from the MEPS database at this URL: https://meps.ahrq.gov/mepsweb/ accessed date 2 September 2024.
